# Peer Facilitators as Core Co-developers of an Online Peer Encouragement Network (OPEN2chat) for Austrian Adolescents

**DOI:** 10.3389/fdgth.2022.833006

**Published:** 2022-06-16

**Authors:** Gloria Mittmann, Susanne Sophie Schmalwieser, Tamara Diendorfer, Beate Schrank, Markus Boeckle

**Affiliations:** ^1^D.O.T. Research Group for Mental Health of Children and Adolescents, Ludwig Boltzmann Society at Karl Landsteiner University of Health Sciences, Vienna, Austria; ^2^Karl Landsteiner University of Health Sciences, Scientific Working Group, D.O.T. - Die offene Tr (The open door), Krems, Austria; ^3^Department of Psychiatry for Adults, University Hospital Tulln, Tulln, Austria

**Keywords:** adolescence, digital intervention, participatory research, co-design, young adults, peer counseling

## Abstract

Adolescence is a crucial developmental time, and it is essential to ensure stable mental health during the transition to adulthood. Peer-to-peer networks seem to be a promising tool to support adolescents during that time. While co-development often concentrates on the end-user, this paper focuses on the peer facilitators of an online peer encouragement network (OPEN2chat), where adolescents can chat with peer facilitators about their problems. We conducted 3 group discussions with 18 peer facilitators after a testing phase to improve the process of these interactions. Thematic analysis was used to analyse the data after transcription. The four main themes were the responsibility of the peer facilitators toward their peers, especially their role of giving advice; the interaction process itself; time management; and technology aspects of the application. Including these stakeholders in the development process empowered the young people, helped eliminate problems with the application, and made the researchers more sensitive toward potential issues and emotions that peer facilitators encounter that may have been missed without a co-development process. Eliminating these problems might also help establish a better environment and support system for the actual end-users.

## Introduction

Adolescence as the transition between childhood and adulthood encompasses significant biological, emotional, physical, social changes and development. Social cognitive and social affective changes are especially notable as peers become increasingly important (1, 2). Adolescents need to manage the transition to adulthood in a healthy way, which can mean many things, from avoiding drugs to stable mental health (3). Positive peer support has been associated with improvements in depressive symptoms and a substantial reduction in suicide attempts (4). It has been shown to serve as a protective factor during interpersonal stress (5). Negative experiences such as bullying or victimization can harm mental health and wellbeing, with risks ranging from drug use to suicidal intentions (6). Many mental health disorders emerge in adolescence, connected to violence and (sexual) risk behavior (7). It is vital to promote healthy behavior in this period and have programs for adolescents who experience or have experienced adverse events and emotions.

A growing body of literature is calling for active investment in the health and wellbeing of adolescents (8, 9) and there is already ample evidence that preventive interventions during adolescence have beneficial effects on physical and mental health outcomes. For example, meta-analyses show positive outcomes for alcohol reduction (10), improving mental health with mindfulness (11), or reducing self-harm (12), showing that there are many possibilities to help and support adolescents overcome or prevent a variety of problems. One thing to consider when designing programs for adolescents is that adolescents' lives are more and more intertwined with the digital world and being online is part of adolescents' everyday lives (13). This has been accelerated by Covid-19 and the related lockdowns, forcing adolescents to experience both their leisure time and school lives almost entirely online. But the pandemic has not only made adolescents be even more prone to spending time in the digital world, it has also led to a strong increase of mental health problems (14). Prevention programs and interventions are not only more vital than ever, they also need to adapt to digitalization by offering online support, and some digital health interventions show promising results regarding the mental health of young people (15). Online programs seem particularly important to allow adolescents to talk about sensitive topics anonymously and confidentially (16), hence giving access to a more diverse group of users as well (17). Comparing online peer-support to face-to-face support has been shown to be equally effective in improving symptoms of users, but additional qualitative findings have shown vast benefits of the online setting in terms of accessibility and feasibility (18).

Looking at Austria specifically, adolescents' mental health service situation is far from perfect: Philipp et al. (19) found that more than 20% of Austrian adolescents between 10 and 18 years old have a diagnosable mental illness. Wagner et al. (20) confirmed this percentage, with the most common mental issues being anxiety or developmental disorders. Ego-syntonic disorders prove to be treated very rarely, with only 25% of patients receiving therapy (21). The supply of mental health professionals and institutions is alarmingly low, with only one child- and adolescent-psychiatrist available for every 30,000 children and adolescents. What is more, individuals seeking mental health support often face additional challenges such as misdiagnoses and stigma (20), which can further be exacerbated by a variety of factors such as ethnic (22) or socio-economic background (23). These findings conclude a need for a broader supply of mental health specialists and institutions for children and young adults (24). Schneidtinger and Haslinger-Baumann (25) constructed a model of Austrian adolescent patients' recovery that shows three main stakeholder groups to be able to function as both barriers and positive “facilitators” of recovery: family, treatment (institutions), and peers. In this study, peers could alleviate mental stress by providing understanding, community, and friendship. Based on this notion, OPEN2chat aims to provide peer support, especially for those who lack support from their peer group in their day-to-day lives and all adolescents who struggle to access professional intervention programs.

OPEN2chat is a web-based application for adolescents co-developed between 2018 and 2021 in close collaboration with adolescents. OPEN2chat was launched on the 1^st^ of December 2021 and connects specifically trained adolescent peer facilitators with peers who want to share psychosocial issues, concerns, and problems anonymously. Peer facilitators undergo training for online and chat counseling. This training includes general information on mental health and mental illness, information on verbal tools, techniques for online counseling, and advice on how to deal with possible psychological stress that could arise throughout the counseling process. The training is composed of input sessions from trainers with vast experience in online counseling and opportunities to gain hands-on experience with OPEN2chat through role play.

However, OPEN2chat is not a counseling tool *per se*, but rather a stigma-free space for exchange with the possibility for young people to articulate their concerns, share opinions and experiences with a peer, and subsequently open up to the idea of professional counseling or therapy if needed. The goals are thus individual and joint reflection and the de-stigmatization of counseling services or therapeutic settings. OPEN2chat can be accessed *via* http://www.open2chat.at and is free of charge. German is currently the only supported language. Peers can register anonymously by using a token and immediately ask their questions to a peer facilitator. Peers can tag their messages with overarching themes (e.g. “school”, “family”, “bullying”, etc.) to allow peer facilitators to handle only those messages they feel familiar with. Peer facilitators can take over newly received messages according to their time capacities and are considered responsible for this contact from then on. If necessary, they can call in supervisors for support. Supervisors are mental health professionals who are available to peer counselors *via* chat, telephone, video call and, if required, in person. They have direct insight into the current chats of the adolescents they are supervising and can intervene if necessary, e.g. when a peer counselor feels mentally burdened by the topic of a conversation. Additionally, supervisors and peer counselors meet regularly in one-on-one sessions to discuss the counsellors' experience with OPEN2chat and their overall mental wellbeing.

To ensure attractiveness for the target group and present the offer in a familiar format to young people, OPEN2chat visually corresponds to a chat tool (like SMS, WhatsApp or Facebook Messenger), e.g., offering standard digital communication tools such as emoticons. At the same time, the communicative synchronicity that is often associated with the services mentioned above is deliberately not provided in OPEN2chat. The peer facilitators are required to react to a message within 72 h at the latest. On the one hand, this is to prevent the peer facilitators from being overburdened, but on the other hand, it also encourages the peers to reflect. The time window between two text tokens in the “chat” can encourage peers to take more time to produce a single token and proceed auto-reflexively. Hence, OPEN2chat can be used in either an a-synchronous e-mail or a classical synchronous chat function.

### Peer Facilitators

To be able to offer this psychosocial support to peers (help seekers) and ideally also benefit from the voluntary work themselves, the counseling young people, who in the case of OPEN2chat are referred to as “peer facilitators,” must above all have the ability to empathize and feel a basic sense of security in their own lives. While neither facilitators in other online support programs like “U25” (26) nor volunteers in the German “Youth-Life-Line” classify the stresses they encountered during counseling as unmanageable (27), the former identify concrete challenges related to their counseling work. Particularly significant here is the confrontation with one's own ability to act, which is perceived as severely limited by the format of anonymous counseling. In connection with this, a lack of answers from the users is described as particularly frustrating. The interviewees in the study of Egli (26) also describe difficulties in experiencing their empathy as limited in some cases, for example when topics trigger adverse emotions in facilitators, or they do not find a typical “wavelength” with those seeking advice. These problems, which are directly related to the counseling process, mean that peer facilitators must draw on a range of coping strategies.

The facilitators at U25 point out the relevance of internal organizational support (26), which supervisors provide at OPEN2chat. While some interviewees state that they resort to external social support, such as friends or family members, others say they rely more on a strict separation of counseling and private life. Thus, it becomes clear that dissociation from the subject matter can be a valuable coping strategy for young facilitators (26). Barbuto et al. (28) also emphasize that counseling topics should be dealt with in the framework provided for this purpose. Facilitators should learn to understand psychosocial issues addressed in the counseling sessions. The facilitators at U25 also consider the training, which takes place before the first counseling sessions, as critical support. During this training, initial counseling sessions are practiced using email examples, information on mental health issues is shared, and trust within the group is strengthened. Since internal support is also seen as the leading resource later in the counseling process, this trust between all participants is particularly relevant. The training can be seen as a basis for avoiding substantial stress in the course of the counseling sessions. Later on, regular expressions of appreciation to the facilitators are important reminders of the support opportunities within the organization (26).

If these sustainable support services are available, facilitators can benefit from the experiences gained during their counseling activities even years later. In total 82% of former peer facilitators of Youth LifeLine state that they can recognize and deal with their crises, 60% accept help from professionals. In total 96% of the respondents think they can also support other people privately under challenging situations; a similar picture emerges concerning education and professional life (27). One reason for these high values can be seen in the positive experience resulting from the analogous relationship between help-seeker and counselor, which stimulates spontaneous self-reflection, trust-building, and self-identification (28).

### Co-development, OPEN2chat, and the Current Study

It is not enough to develop offers for vulnerable populations such as adolescents from an external development team or mental health professionals; it is also a vital point to include the people in the process and evaluation of such offers and give them a voice (29). Participatory research is an approach that includes people in various stages of the research process. It gives them the power to be involved and make and inform critical decisions, hence breaking the hierarchy of the researcher being the “expert” by making the population co-researchers (30, 31). Co-development for mental health and wellbeing programs has recently received increased interest (32, 33). Participatory development can range from consultative to collaborative (32). Either version of involving young people, in particular, does not only have beneficial effects for the research process but also on the empowerment and capacities of the young people involved (34), leading to a positive impact on mental health and wellbeing in itself (35). Bevan Jones et al. (32) conclude that emerging studies involve young people and specific subgroups in co-design. Still, they state that specific subgroups like younger children and those with learning disabilities and specific difficulties are under-represented and have particular needs and preferences that might not be acknowledged in current approaches. Finally, in their perspective, research is required to look at technologies' developmental and implementation phases. The co-design practices might then become the new benchmark for how digital technologies of high quality are developed. Furthermore, it is essential to include the relevant population in scientific development and describe this process to evaluate the impact properly (36).

Mainly, peer-to-peer support systems' co-development focuses on end-users' perspectives of such platforms. While this is undoubtedly vital for the success of these platforms, we argue that including the peer facilitators in the co-development process will likely also influence the experience for the end-user. For example, in a literature review about peer support mental health interventions, co-design and development mainly were done with peers, i.e., those seeking support (37). In the current paper, we describe the process and influence of co-development with peer facilitators (i.e., the people who provide support) of the online peer encouragement network OPEN2chat. The webtool offers adolescents an anonymous platform where they can talk to and share their problems with specifically trained adolescent peer facilitators. These peer facilitators have been involved at various stages of the process and have co-developed the platform from users and facilitators' perspectives. First, they were involved in the needs analysis in 2018 (investigating the needs of adolescents in Austria regarding their mental wellbeing) that resulted in the establishment of a co-development team in 2019. This team was constantly involved in all phases of the development until the prototype of the web application was established in 2020. After installing the web-tool proto-type, additional adolescents and young adults who had shown interest in peer facilitators' roles were invited to test the current version. We describe their primary input, ideas, and concerns by analyzing 3 group discussions after a single testing session of the tool for each group. Subsequently, the results of the first use of the prototype are presented. We also discuss how their input was implemented in further development.

## Methods

### Population of Peer Facilitators

We actively recruited testing peer facilitators at secondary, upper secondary, and vocational schools and students at bachelor's degree programs in, e.g., psychology and social work between 16 and 21 years old. We recruited in the respective schools *via* social media and printed flyers in the year groups of the study programs and schools and asked those involved to tell friends, family members, and the classroom to join the test development group. Additionally, we promoted the program *via* social media in parent groups. We recruited for three sessions of test peer-facilitators in 2021. Adolescents were invited to test the OPEN2chat prototype and were subsequently informed about our scientific aims and data collection methods. Before the testing period, all adolescents were interviewed by a research team member to assess whether they could handle the time expenditure and potential emotional stress of using OPEN2chat. Thus, all adolescents interested in testing OPEN2chat and who passed the interview stage were included in the sample. We did not need to exclude any subjects from participation.

The sample consisted of a total of 21 adolescents (18 female, 3 male) between the ages of 16 and 22, who were interviewed in groups of 6, 8, and 7 according to their time of participation (either February, June or September 2021). Out of the 21 participants, 20 were university students of various Bachelor's programs in Austria, while one was a student in secondary school. One of the previous need analysis participants also was a test peer facilitator. Apart from this participant, none of the adolescents had any previous contact with the OPEN2chat project.

### Interview Procedure and Structure

Each group participated in two online sessions, with 2 weeks testing the online application in between. The first session consisted of presenting the tool. It defined pairs of facilitators that alternated the role of test facilitator and test peers (i.e., the person who seeks help). Individuals stayed in one role for 1 week. After the test phase, a group discussion took place. Each session had two or three moderators with one designated discussion leader. Session duration was between 60 and 120 min and followed a predefined interview guideline. This guideline included questions on technical aspects of the communication and questions about the communication process and the individual experiences of each test peer facilitator. The ethics committee approved the study (EKNr: 1070-2020), and informed consent was required from all participants.

### Analysis

Group discussions were transcribed *via* a word processing application with the following transcription rules: Dialectal utterances were largely transcribed as standard Austrian German, some exceptions being diminutives (“ein bissl”) or contractions (e.g. “ich kann's”). Phonetic tokens not carrying a lexical meaning (e.g. “ahm”) were not transcribed. Acoustically unintelligible utterances were marked with “[unverständlich]”/“[unintelligible]”. While the researchers are identifiable with their first names in the transcribed files, participants were assigned numbers according to the order they first spoke during the recording.

Qualitative analysis was conducted *via* QSR International's Nvivo (38). After transcription, discussion notes were transferred to the software. We analyzed the data using thematic analysis (39) because it can describe the participants' reality and experiences without pre-existing theoretical assumptions. All data was coded by one coder (S.S.S) and reviewed by a second coder (G.M.). The ongoing discussion between all authors resolved discrepancies, which led to the restructuring, deletion, and collapse of some categories. Saturation was reached after the third focus group, as no new themes emerged. The authors translated quotes that were used in this article.

## Results

**Figure 1:**
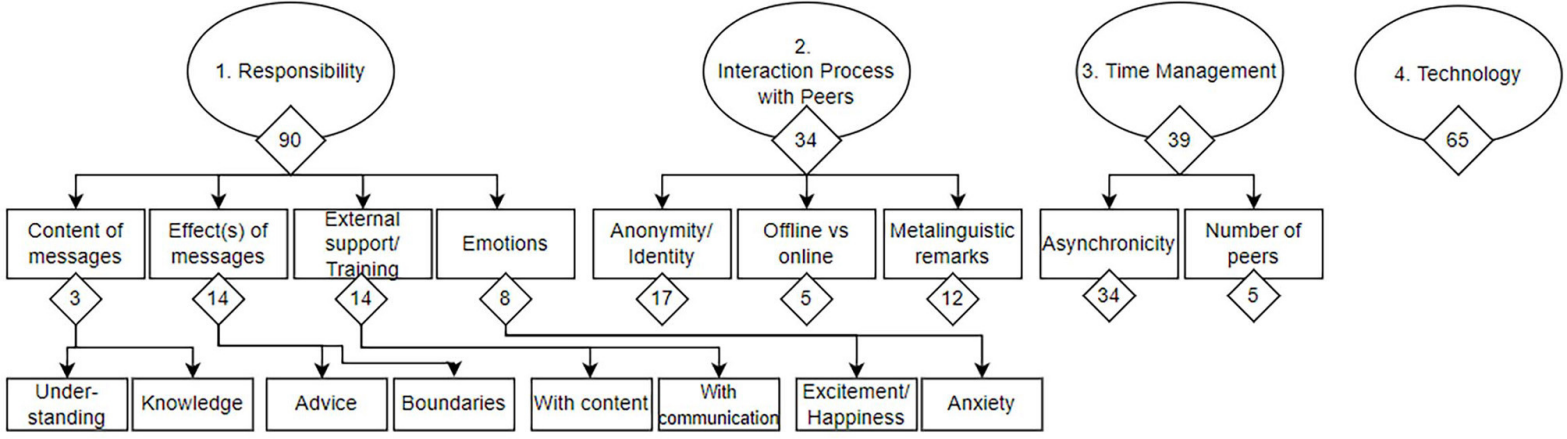
Coding tree of themes and subthemes. The number of coded sequences per theme can be found below each node.

Four categories were the main themes of the participating peer facilitators (i.e. people who provide support): (1) Responsibility covers concerns about how to handle the sensible topics brought on by the peers; (2) The interaction process includes peer facilitators' thoughts about the whole communication process with peers; (3) Time management, including the timing between interactions and the number of peers; and (4) Technology, which covers all problems and ideas on a technical developmental level. Categories were further coded into sub-themes (see Figure 1).

### Responsibility of Peer Facilitators

The first theme describes peer facilitators' thoughts about the (self-perceived) responsibility when interacting with peers and helping them with their problems. For peer facilitators, who are mostly teenagers themselves and not trained psychologists, this seems to be the theme related to the highest level of anxiety. Concerns related to the contents of messages, the effects of messages on peers, external support, training, and emotions.

#### Content(s) of the Messages

The contents of the messages were addressed frequently during the group discussions. Peer facilitators identified topics about which they already had knowledge through their own experiences to be easier to relate to. On the other hand, a lack of knowledge about an issue led some facilitators to feel overwhelmed (e.g., when asked for advice on a peer's parents' divorce while never having experienced a similar issue). This emphasizes the importance of peer facilitators choosing peers according to their problems to facilitate helpful advice and ensure that facilitators feel confident and secure. Other than just knowing the problem, uncertainty could arise because the facilitator did not have enough information, was unsure about the topic, or could not think of any advice for the given situation. Peer facilitators agreed that there were different difficulty levels regarding the severity and the content of the problem, which could be subjectively more complex or more straightforward. For example, “school” was seen as a relatively simple topic, though some needed time to emphasize since it has been some time since they had attended school. A topic that was perceived as more difficult was problems with other friends or peers, which involved people who were not part of the conversation.

“*And yeah, the person also didn't know how to manage at school, when the person no longer has any friends. And yeah, I think I would definitely say that it is easier for me or that the topic is easier here. That it can help more directly than with the other topic with the friend and much more.” (2-06)*

In this context, it should be noted that many facilitators felt the urge to provide their peers with pieces of advice in their first few messages and feared seeming intrusive when asking too many questions. Some voiced concern that they did not feel like they were able to tell the other person what to do. The following quote shows how a peer facilitator struggled because they did not feel confident to give concrete advice:

“*I can understand that well […], you want to say something, but […] I somehow had too little information to really say / [what] the person should do now and you don't want to say: okay that would be good, and that would be wrong or that would be bad or that would be good[…]” (1-03)*

The juxtaposition of “good”/“bad”/“wrong” illustrates that the facilitator, on the one hand, feels urged to give specific advice, i.e., to directly react to the content of the user's message with ideas or suggestions on how to solve their issue. On the other hand, however, they do not want to do this due to a lack of knowledge about the user's situation. This ambivalence might lead to a conflict of interests that the peer facilitators need to resolve to proceed with the counseling process.

#### Effect(s) of the Message on Peers

A dominant topic throughout all three discussions was the possible effects of the peer facilitators' messages on the recipients. Some concerns were, for instance, the extent to which said messages could help the peers with their issues, how a welcoming environment could be created within the chat, how not to overstep boundaries when asking questions or giving advice and how to show empathy *via* written text messages. Peer facilitators found it challenging to find the line between asking questions and not being too intrusive and between being empathetic but still professional, for example, when disagreeing with the peer's parents.

“*[…] it was often the case that when I suggested something, the response was “I can't do that because of my parents”, and I found it very difficult not to say, “I don't think it's cool that your parents don't do that”, i.e. not to condemn the person's parents […]” (3-04)*

#### External Support and Training

Participants described scenarios where they would have liked to seek external support from trained supervisors. These scenarios mostly revolved around receiving messages that proved problematic in their content. Additionally, some adolescents mentioned a need for professional digital and written communication skills to feel more secure.

“*And then, as I said, I was stuck for a while. And maybe, if it had gone in that direction somehow, I would have asked [a supervisor] at some point. That would have been the only situation where I would have asked for more ideas, creatively speaking. Somehow, I probably would have asked. Exactly. So just inventiveness, in that respect.” (2-01)*

“*I just think that it would be cool if you gave us a certain basic structure at this seminar for asking questions, but I guess you already have planned something.” (3-03)*

#### Emotions

Most peer facilitators agreed that the excitement and anxiety were worst during the first interaction but diminished over time.

*So I definitely have the feeling that this just gets easier and certainly works better the more often you do it - I'm also sure when you get more used to this medium, when you write about such things or such problems. (1-03)*

An initial feeling of being overwhelmed also accompanied this. On the other hand, they also mentioned positive emotions, especially a happy excitement when receiving an email and satisfaction after successfully replying to a peer's message.

“*But it definitely feels good when you write a message and, as I wrote, you're somehow satisfied with it, where you have the feeling that you've somehow got it across well, so that's already / I think that feels good somehow, even when it's sent, and that's somehow so (…) and you have the feeling that you've somehow got to the heart of it then / it's a good feeling anyway.” (1-03)*

### Interaction Process With Peers

#### Anonymity and Identity

Participants discussed to what extent their and their partners' anonymity influenced their communication strategies when chatting *via* OPEN2chat. Some issues mentioned in connection to the peers' and peer facilitators' anonymity were a greater struggle to understand the peers' background and thus their problems. Another point was that communication with an anonymous other felt “strange”. They further mentioned ideas on how personal identity can or could be constructed within this medium, e.g., by providing their communication partners with basic information about their gender, age, or hobbies.

The test peer facilitators acknowledged acquiring information about the peer and their respective issues as their primary aims when composing the first few messages within the chat. Due to the anonymity, the first goal would be to understand the personality, the writing style, and how much is written between the lines.

“*So I think you have to be more careful than when you know a person personally because it's difficult to know exactly what will help the person. There are people for whom it is a great help if you are simply very direct and say what strikes you, and others are more likely to be offended or offended if you are open, and so you have to be more careful overall.” (2-02)*

“*But I would definitely, what we already do anyway, that you can choose the age and which gender, so the criteria you can choose. Maybe hobbies, but that you somehow, possibly also pictures somehow, so I don't exclude that now either. But I would think it would be good that way.” (3-06)*

#### Offline vs. Online

Some participants voiced that the online environment caused additional challenges for peer facilitators, such as a lack of non-verbal elements of communication. They felt that it was harder to be empathetic due to the online anonymity, as you cannot see how the peer receives the message. They also argue that you do not have to filter what you say as much as during an online message in a face-to-face conversation because the message should not be too long. On the other hand, peer facilitators found the spatial distance and anonymity reasonable to think about the problem and their answers.

“*So it's a bit difficult to find a middle ground, because you don't know how it will come across at the other end, because when you're sitting in front of the person, you can convey a lot that can't be expressed in words and that's kind of difficult to say - how do I convey it in such a way that it's still like / so that in the end it's just my (…) what I have from the information I have so far and how I think I would deal with it.” (1-03)*

In this quote, a peer facilitator describes their struggle with a lack of non-verbal information in the chat format. They express their aim to react accordingly to the information given verbally by the user, without compensating the lack of non-verbal cues by inferring subtext to the messages that the user might not have intended.

#### Meta-Linguistic Remarks

When discussing the effects and contents of their messages, the test peer facilitators also reflected on the language they used, e.g., in terms of wording or structure.

“*Then I also tried to summarize things or to keep them as open as possible, or, I think I wrote once, it is important that I know this more precisely in order to be able to help you. Yeah, to kind of explain why I do it that way.” (2-05)*

“*Because I just find it a bit difficult in the beginning when chatting, that you have to collect your thoughts, your thoughts a bit and then really have to pay attention to, okay, what exactly do you want to say now, what is the most important thing. Yes, I think in the chat it's better not to write too much anyway, so that it's clear.” (2-06)*

### Time Management

#### Asynchronicity

Mostly, peer facilitators agreed that the asynchronicity that comes with the online environment makes it easier for them to reply to the problem. Taking some time to think about their response, especially after receiving the first message, helped reduce anxiety in the peer facilitators.

“*So for me, when the first request is made, I would like to be able to take my time and somehow think about it for a day or two. And you usually get a relatively long text at the beginning, and then you will answer in a relatively long text, and if there are follow-up questions or certain follow-up questions, then it can perhaps be fine that you answer relatively directly, that a conversation arises from it and that it are not somehow certain questions that are aimed at something specific.” (1-04)*

“*And then it wasn't so easy for me/so I spent a really long time making improvements on the message and a super long message/I also had a hard time giving my opinion, but somehow coming off too strong, too (…) too pushy with my opinion or something. So I fiddled around for a long time at the beginning, but I was similar to B and then it got better somehow and the next messages were easier.” (1-03)*

A significant theme of peer facilitators during all group discussions was time management: While wanting to take enough time composing a message to accommodate their schedules and sufficiently reflect on the peer's question or problem, the peer facilitators did not want to keep their peers waiting for too long. Some preferred to reply within a 24- or 48-h window to be still familiar with the content of the chat when composing the next message. The following quote illustrates how a peer intentionally used a longer time frame to write a thoughtful response:

“*But I also believe that it is important at the beginning just/so I also believe that it is important, to basically also have this time and to say, okay, you can think about it, because the in the chat procedure now just already shown who there already once (unintellig.) to pay attention that one doesn't communicate anything in a wrong way and therefore I also find it important that one just also knows, okay, one can think it over for 1 day or so.” (1-03)*

At the same time, a different peer facilitator preferred answering immediately after receiving a message. This variety of opinions shows the importance of a tool fit for various communication styles.

“*So it's easier for me if I answer straight away because then I'm simply confronted with the problems for the first time, and then my answer immediately contains my first helper thoughts, so to speak.” (1-05)*

#### Number of Peers

Another topic concerned the number of peers/messages that peer facilitators could handle. There were different opinions about that, depending on the problem and personal time availability. Some said that around three other peers should be manageable for them as peer facilitators to handle at the same time.

“*So I'm very quick with answers and yes / think so because simply at least three people at the same time would be possible as well and I could write to them because it's easy for me.” (1-05)*

#### Technology

Even though technology was one of the most frequently discussed themes in our sample, we will only briefly describe the outputs. Most of the time, technological features are very straightforward and do not call for in-depth discussion. Instead, their implementation depends on the responsible program developers' time, money, and competencies. In our case, as a result of the group discussions, we focused on implementing the two main improvements mentioned below, which have already been implemented since the group discussions.

Participants discussed a wide range of technological features and the prototype they had tested. They described problems they had encountered, aspects they deemed neutral or positive and their wishes for improvement and adaptation of the tool. The two main ideas that the developers of OPEN2chat implemented were the function of the Enter key to start a new paragraph instead of sending the composed message right away and the improvement of the efficiency of e-mail notifications (as some have not received any).

“*And on the mobile phone, the problem was that the messages were displayed in such a way that you couldn't read them, exactly, and the last answer was also covered by the chat bar, so I had some problems there.” (3-03)*

“*The only point I also want to make is this Enter key for sending. Sometimes it was annoying, especially when you wanted to go into more than one thing and needed a paragraph.” (2-05)*

## Discussion

This paper describes the importance of including peer facilitators in the co-development of an online peer support tool to develop and improve OPEN2chat. Three groups of peer facilitators conducted a 2-week testing session of the tool, followed by an online group discussion. Four main themes were discussed during those sessions. First, peer facilitators' concerns about their responsibility of giving advice; second, the online interaction process with the peers; third, the time management of the tool; and fourth, technology.

It is difficult for trained psychologists or researchers to understand the fears and concerns of being an untrained peer facilitator without their direct input. Yet, feeling anxiety when interacting with peers as a peer facilitator might lead to a negative experience for the peer facilitator and the peer. This underlines the importance of including all stakeholders in the co-development process, especially if the group is directly using the platform with the peers. We found that peer facilitators' main point of anxiety was during the first contact with their peers, which is also discussed in previous studies (e.g. (26)). This relates primarily to peer facilitators' insecurity about writing the first message. Depending on the topic of the problem, this is perceived as more accessible or more complex and necessarily needs high-quality training programs (26).

The online environment and the resulting anonymity can heighten the anxiety (40), though it also bears advantages such as having time to think about an appropriate reply. The stress related to writing the first message and the responsibility of helping another person with their problems may be reduced if new peer facilitators get more intense help from a supervisor during their first peer interaction. As facilitators in our sample had not done this kind of helping before, they might be more confident after a few different peers to communicate with. Helping them with their peer might ease them into the process and make them more relaxed when writing with their future peers. It has also been shown that peers' counseling-based training affects the peer relationship and the quality of the support given (41). Therefore, our training can potentially improve the confidence of our peer facilitators.

Some discussion points were more straightforward to resolve than others. Time management and especially the timing between the first message from a peer and the first reply was a central discussion point for the peer facilitators. Yet, there was no consensus about the correct restrictions/freedoms. Some preferred shorter periods to be familiar with the problem, some selected more extended periods to think about the issue in-depth. Time management and overload seem to be somewhat subjective problems, so peer facilitators should have as much freedom as possible when deciding how many peers they want to accept at the same time. Other online peer support platforms have stated that creating guidelines and ground rules for all facilitators in advance has benefited everyone included. Therefore, these give a framework and confidence and guidance (42).

It is important to note that technology was the most prominent category in our sample. It became apparent that by using an interview guideline that was rather open-questioned, group discussion participants tended to talk about more obvious topics such as technical features. It seems logical that these topics are easier to think and talk about. Following this observation, it seems desirable to prepare a more detailed interview guideline before the group discussion. In that case, even if the debate is supposed to be very open (as it can have advantages to let participants speak freely), it gives the group discussion leader the possibility to steer the group to less obvious or “harder” topics if necessary.

Peer support interventions/peer-to-peer networks seem to show positive results concerning feasibility, acceptability, and effectiveness (37). We know that adolescents are confident about interacting online in a digital environment (43). Yet, the specific setting of a support tool still poses the potential for anxiety for the peer facilitators due to the responsibility they feel when advising other unknown adolescents. The main aim is to improve how confident the peer facilitators feel, especially during the first interaction. This can be achieved by giving them the freedom to choose between different peers/topics and offering support by a senior psychologist/researcher during this time. The fact that the support system is embedded in a digital environment bears both advantages, such as having more time to formulate an appropriate response, and disadvantages, such as lack of non-verbal communication cues. Adolescents nowadays are digital natives and used to navigating through an online space. Yet, intimate topics are usually discussed in an offline environment (44). Due to missing facial cues in an anonymous online conversation, it is harder to know another person's feelings (45).

### The Importance of Co-development and the Future of OPEN2chat

Including young people seems to be especially important to develop mental health applications as the taste and interests and mental health problems differ between adults and young people, which makes it harder for adults to design age-appropriate programs (32). By including potential peer facilitators in the development process from the beginning and addressing their concerns in an ongoing process, peers, peer facilitators, and involved researchers/practitioners will benefit from the web application, which will undergo constant adaptations (32, 33). This paper describes the involvement of one group of stakeholders, namely the peer facilitators. Co-development is an ongoing process that involves all relevant stakeholders.

As limitations, we have to report typical problems of co-development (32). Our sample was relatively small and might not represent the population in general. Additionally, participating adolescents and young adults might underly self-selection because they are more interested in mental health issues. This is also reflected in the preponderance of women in our sample, which was constituted by only 14% of male adolescents. This low percentage is not surprising as there is a general predominance of women in the health care sector (46).

Future development of the tool will show whether the end-users of the device will report necessary adaptations. Co-development with peer facilitators (and not end-users) is a limitation and strength of this study. While it is not enough to only co-develop with peer facilitators, it also provides an essential step to including all relevant stakeholders. Even if we are not calling the peer facilitators “end users,” they will use the platform just as much or even more than the peers in the end. Additionally, co-development and evaluation of the platform with peers will be done in the future.

As of November 2021, hosting the tool has been overtaken by the Caritas der Diözese St. Pölten and Caritas der Erzdiözese Wien, and the first group of peer facilitators has completed their training. Many papers and reviews describe low user engagement with digital intervention as a challenge that a rigorous co-design process might reduce through a positive relationship with users and a higher quality of the application (47). Initial user engagement for OPEN2chat (which launched in December 2021) was positive both with peers and peer facilitators, which shows the first evidence for a successful implementation. Yet, we cannot say if this is related to the user engagement throughout development, as we did not specifically address the impact of co-development as an outcome. This is a relevant problem in the literature on participatory research (33). We plan to qualitatively evaluate the impact of our co-development by addressing this question in the ongoing collaborative work with peer facilitators, who have been part of the development and are still part of the team that uses the web application.

It should be noted that we aim to continue the co-development process after the initial launch in December 2021. We plan to adapt and improve OPEN2chat based on scientific evaluation centered around adolescent users on both ends of the chat. Using quantitative and qualitative methods, we aim to collect data on the effectiveness of the intervention for peers and data on the wellbeing of peer facilitators. Based on these findings, which will be communicated to the responsible software engineers, we hope to ensure the lasting usability and appeal of OPEN2chat for our target group.

## Conclusion

In conclusion, co-development is vital for developing online tools, especially for children and adolescents. Including all relevant stakeholders such as peers and peer facilitators add to the end product's quality. Giving that group a voice can only improve the design and content of such a tool, and we argue that co-development should be implemented whenever possible. As a next step, we will evaluate the efficacy of the application and will continue to improve based on the evaluation and implementation of our formative research approach. Only *via* such broad co-development and the following empirical assessment of the program's efficacy will it be possible to differentiate between co-developed programs and those that are not.

## Data Availability Statement

The raw data supporting the conclusions of this article will be made available by the authors, without undue reservation.

## Ethics Statement

The studies involving human participants were reviewed and approved by Commission for Scientific Integrity and Ethics of the Karl Landsteiner Private University for Health Sciences (EKNr: 1070-2020). Written informed consent from the participants' legal guardian/next of kin was not required to participate in this study in accordance with the national legislation and the institutional requirements.

## Author Contributions

MB, GM, SS, and BS conceived of the presented idea. SS and MB interviewed the participants. SS and GM transcribed the audio files and analyzed the data. MB, SS, TD, and GM wrote the first draft of the manuscript. All authors discussed the results and contributed to the final manuscript and authors contributed to the article and approved the submitted version.

## Funding

This work was supported by the Ludwig Boltzmann Society and the Karl-Landsteiner University of Health Sciences.

## Conflict of Interest

The authors declare that the research was conducted in the absence of any commercial or financial relationships that could be construed as a potential conflict of interest.

## Publisher's Note

All claims expressed in this article are solely those of the authors and do not necessarily represent those of their affiliated organizations, or those of the publisher, the editors and the reviewers. Any product that may be evaluated in this article, or claim that may be made by its manufacturer, is not guaranteed or endorsed by the publisher.

## References

[1] Crone EA Dahl RE. Understanding adolescence as a period of social–affective engagement and goal flexibility. Nat Rev Neurosci. (2012) 13:636–50. 10.1038/nrn3313 22903221

[2] Lam CB McHale SM Crouter AC. Time with peers from middle childhood to late adolescence: developmental course and adjustment correlates. Child Dev. (2014) 85:1677–93. 10.1111/cdev.12235 24673293 PMC4107039

[3] Taghizadeh Moghaddam H Bahreini A Ajilian Abbasi M Fazli F Saeidi M. Adolescence Health: the needs, problems and attention. Int J Pediatr. (2016) 4:1423–38. 10.22038/ijp.2016.6569

[4] Czyz EK Liu Z King CA. Social connectedness and one-year trajectories among suicidal adolescents following psychiatric hospitalization. J Clin Child Adolesc Psychol. (2012) 41:214–26. 10.1080/15374416.2012.651998 22417194 PMC3742020

[5] Mackin DM Perlman G Davila J Kotov R Klein DN. Social support buffers the effect of interpersonal life stress on suicidal ideation and self-injury during adolescence. Psychol Med. (2017) 47:1149–61. 10.1017/S0033291716003275 27995812

[6] Moore SE Norman RE Suetani S Thomas HJ Sly PD Scott JG. Consequences of bullying victimization in childhood and adolescence: A systematic review and meta-analysis. World J Psychiat. (2017) 7:60. 10.5498/wjp.v7.i1.60 28401049 PMC5371173

[7] Costello A Naimy Z. Maternal, newborn, child and adolescent health: challenges for the next decade. Int Health. (2019) 11:349–52. 10.1093/inthealth/ihz051 31529111

[8] Dahl RE Allen NB Wilbrecht L Suleiman AB. Importance of investing in adolescence from a developmental science perspective. Nature. (2018) 554:441–50. 10.1038/nature25770 29469094

[9] Sheehan P Sweeny K Rasmussen B Wils A Friedman HS Mahon J. Building the foundations for sustainable development: a case for global investment in the capabilities of adolescents. Lancet. (2017) 390:1792–806. 10.1016/S0140-6736(17)30872-3 28433259

[10] Tanner-Smith EE Lipsey MW. Brief alcohol interventions for adolescents and young adults: A systematic review and meta-analysis. J Subst Abuse Treat. (2015) 51:1–18. 10.1016/j.jsat.2014.09.001 25300577 PMC4346408

[11] Kallapiran K Koo S Kirubakaran R Hancock K. Effectiveness of mindfulness in improving mental health symptoms of children and adolescents: a meta-analysis. Child Adolesc Ment Health. (2015) 20:182–94. 10.1111/camh.12113 32680348

[12] Ougrin D Tranah T Stahl D Moran P Asarnow JR. Therapeutic interventions for suicide attempts and self-harm in adolescents: systematic review and meta-analysis. J Am Acad Child Adolesc Psychiat. (2015) 54:97–107.e102. 10.1016/j.jaac.2014.10.009 25617250

[13] Tsitsika AK Tzavela EC Janikian M Olafsson K Iordache A Schoenmakers TM. Online social networking in adolescence: patterns of use in six European countries and links with psychosocial functioning. J Adolesc Health. (2014) 55:141–7. 10.1016/j.jadohealth.2013.11.010 24618179

[14] Singh S Roy MD Sinha CPTMK Parveen CPTMS Sharma CPTG Joshi CPTG. Impact of COVID-19 and lockdown on mental health of children and adolescents: a narrative review with recommendations. Psychiatry Res. (2020) 293:113429. 10.1016/j.psychres.2020.113429 32882598 PMC7444649

[15] Hollis C Falconer CJ Martin JL Whittington C Stockton S Glazebrook C. Annual research review: digital health interventions for children and young people with mental health problems–a systematic and meta-review. J Child Psychol Psychiat. (2017) 58:474–503. 10.1111/jcpp.12663 27943285

[16] Grové C Reupert A Maybery D. The perspectives of young people of parents with a mental illness regarding preferred interventions and supports. J Child Fam Stud. (2016) 25:3056–65. 10.1007/s10826-016-0468-827782017

[17] Barak A Grohol JM. Current and future trends in internet-supported mental health interventions. J Technol Hum Serv. (2011) 29:155–96. 10.1080/15228835.2011.616939

[18] Drysdale MT McBeath ML Callaghan SA. The feasibility and impact of online peer support on the well-being of higher education students. J Mental Health Train Educ Pract. (2021) 3:206–17. 10.1108/JMHTEP-02-2021-0012

[19] Philipp J Zeiler M Waldherr K Nitsch M Dur W Karwautz A. The mental health in austrian teenagers (MHAT)-study: Preliminary results from a pilot study. Neuropsychiatrie. (2014) 28:198–207. 10.1007/s40211-014-0131-9 25429889

[20] Wagner G Zeiler M Waldherr K Philipp J Truttmann S Dür W. Mental health problems in Austrian adolescents: a nationwide, two-stage epidemiological study applying DSM-5 criteria. Eur Child Adolesc Psychiat. (2017) 26:1483–99. 10.1007/s00787-017-0999-6 28540609 PMC5701961

[21] Fuchs M Karwautz A. Epidemiologie psychischer störungen bei kindern und jugendlichen: eine narrative übersichtsarbeit unter berücksichtigung österreichischer daten. Neuropsychiatrie. (2017) 31:102. 10.1007/s40211-017-0238-x 28853032

[22] Gutmann MT Aysel M Özlü-Erkilic Z Popow C Akkaya-Kalayci T. Mental health problems of children and adolescents, with and without migration background, living in Vienna, Austria. Child Adolesc Psychiat Mental Health. (2019) 13:35. 10.1186/s13034-019-0295-y 31528201 PMC6737609

[23] Philipp J Zeiler M Waldherr K Truttmann S Dür W Karwautz AFK. Prevalence of emotional and behavioral problems and subthreshold psychiatric disorders in Austrian adolescents and the need for prevention. Soc Psychiatry Psychiatr Epidemiol. (2018) 53:1337. 10.1007/s00127-018-1586-y 30159723 PMC6267139

[24] Huemer J Völkl-Kernstock S Yee A Bruckner T Skala K. Die Boje“: inanspruchnahme eines niedrigschwelligen ambulanten Therapieangebotes für traumatisierte Kinder und Jugendliche in Österreich. Neuropsychiatrie. (2016) 30:32. 10.1007/s40211-016-0178-x 26961161 PMC4799272

[25] Schneidtinger C Haslinger-Baumann E. The lived experience of adolescent users of mental health services in Vienna, Austria: A qualitative study of personal recovery. J Child Adolesc Psychiatr Nurs. (2019) 32:121. 10.1111/jcap.12245 31310432

[26] Egli N. Herausforderungen von Peer-Beratenden in der Online-Suizidprävention. Ergebnisse einer qualitativen Forschungsarbeit über die Beratungstätigkeit bei [U25]. E-Beratungsjournal. (2015) 11:24–35.

[27] Weinhardt M. Peerberatung im internet – ausgewählte studienergebnisse. E-Beratungsjournal. (2015) 11:3–10.

[28] Barbuto R Biggeri M Griffo G. Life project, peer counselling and self-help groups as tools to expand capabilities, agency and human rights. Alter. (2011) 5:192–205. 10.1016/j.alter.2011.05.007

[29] Bonevski B Randell M Paul C Chapman K Twyman L Bryant J. Reaching the hard-to-reach: a systematic review of strategies for improving health and medical research with socially disadvantaged groups. BMC Med Res Methodol. (2014) 14:1–29. 10.1186/1471-2288-14-42 24669751 PMC3974746

[30] Abma T Banks S Cook T Dias S Madsen W Springett J. Participatory Research For Health and Social Wellbeing. Cham: Springer International Publishing. (2019). 10.1007/978-3-319-93191-3

[31] Cornwall A Jewkes R. What is participatory research? Soc Sci Med. (1995) 41:1667–76. 10.1016/0277-9536(95)00127-S 8746866

[32] Bevan Jones R Stallard P Agha SS Rice S Werner-Seidler A Stasiak K. Practitioner review: Co-design of digital mental health technologies with children and young people. J Child Psychol Psychiat. (2020) 61:928–40. 10.1111/jcpp.13258 32572961 PMC7611975

[33] Orlowski SK Lawn S Venning A Winsall M Jones GM Wyld K. Participatory research as one piece of the puzzle: a systematic review of consumer involvement in design of technology-based youth mental health and well-being interventions. JMIR Human Factors. (2015) 2:e4361. 10.2196/humanfactors.4361 27025279 PMC4797690

[34] Cahill C. Doing research with young people: Participatory research and the rituals of collective work. Children's Geograph. (2007) 5:297–312. 10.1080/14733280701445895

[35] Oliver KG Collin P Burns J Nicholas J. Building resilience in young people through meaningful participation. Australian e-J Advance Mental Health. (2006) 5:34–40. 10.5172/jamh.5.1.34

[36] Maheu-Cadotte M Dubé V Cossette S Lapierre A Fontaine G Desch M. Involvement of end users in the development of serious games for health care professions education: Systematic descriptive review. JMIR Serious Games. (2021) 9:E28650. 10.2196/28650 34129514 PMC8414295

[37] Fortuna KL Naslund JA LaCroix JM Bianco CL Brooks JM Zisman-Ilani Y. Digital peer support mental health interventions for people with a lived experience of a serious mental illness: systematic review. JMIR Mental Health. (2020) 7:e16460. 10.2196/16460 32243256 PMC7165313

[38] QSR International Pty Ltd (2018). NVivo Qualitative Data Analysis Software. In (Version 12) QSR International Pty Ltd. Available online at: https://www.qsrinternational.com/nvivo-qualitative-data-analysis-software/home

[39] Braun V Clarke V. Using thematic analysis in psychology. Qual Res Psychol. (2006) 3:77–101. 10.1191/1478088706qp063oa

[40] Weinstein EC Selman RL. Digital stress: adolescents' personal accounts. New Media Society. (2016) 18:391–409. 10.1177/1461444814543989

[41] Lekka F Efstathiou G Kalantzi-Azizi A. The effect of counselling-based training on online peer support. Br J Guid Counsel. (2015) 43:156–70. 10.1080/03069885.2014.959472

[42] Watson E Lambert M Machin K. Peer support training: values, achievements and reflections. Mental Health Pract. (2016) 19. 10.7748/mhp.19.9.22.s20

[43] Feierabend S Plankenhorn T Rathgeb T. KIM-Studie 2016. Kindheit, Internet, Medien. Basisuntersuchung zum Medienumgang 6- bis 13-jähriger. (2016) 1:1–92.

[44] Mittmann G Woodcock K Dörfler S Krammer I Pollak I Schrank B. “TikTok Is My Life and Snapchat Is My Ventricle”: a mixed-methods study on the role of online communication tools for friendships in early adolescents. J Early Adolesc. (2021) 42:172–203. 10.1177/02724316211020368

[45] Nesi J Choukas-Bradley S Prinstein MJ. Transformation of adolescent peer relations in the social media context: Part 1—A theoretical framework and application to dyadic peer relationships. Clin Child Fam Psychol Rev. (2018) 21:267–94. 10.1007/s10567-018-0261-x 29627907 PMC6435354

[46] Tay PKC Ting YY Tan KY. Sex and care: The evolutionary psychological explanations for sex differences in formal care occupations. Front Psychol. (2019) 10:867. 10.3389/fpsyg.2019.00867 31057471 PMC6478767

[47] Thabrew H Fleming T Hetrick S Merry S. Co-design of eHealth interventions with children and young people. Front Psychiatry. (2018) 9:481. 10.3389/fpsyt.2018.00481 30405450 PMC6200840

